# Influence of subnational contextual factors on demand for family planning satisfied by modern methods: a multilevel approach in 46 surveys from low- and middle-income countries

**DOI:** 10.1136/bmjopen-2025-098980

**Published:** 2025-11-23

**Authors:** Natália P Lima, Cauane Blumenberg, Franciele Hellwig, Aluisio J D Barros, Fernando C Wehrmeister

**Affiliations:** 1 International Center for Equity in Health, Universidade Federal de Pelotas, Pelotas, Rio Grande do Sul, Brazil; 2 Instituto Nacional de Saúde da Mulher, da Criança e do Adolescente Fernandes Figueira, Fundação Oswaldo Cruz, Rio de Janeiro, Brazil; 3 Causale Consultoria, Pelotas, Rio Grande do Sul, Brazil

**Keywords:** Health Equity, EPIDEMIOLOGY, PUBLIC HEALTH

## Abstract

**Abstract:**

**Objectives:**

Understanding contextual drivers of family planning is crucial for designing effective, context-specific policies and programmes. This study aimed to assess (1) the extent to which province-level contextual factors are associated with both coverage and wealth-related inequalities in demand for family planning satisfied by modern methods (mDFPS) across provinces in low- and middle-income countries (LMICs), and (2) whether these factors influence mDFPS at women’s level.

**Design:**

Observational study using multilevel modelling at both ecological and individual levels.

**Setting:**

We analysed data from Demographic and Health Surveys between 2011 and 2022 in 46 LMICs.

**Participants:**

Ecological analysis included 621 provinces. Individual-level analysis included 302 493 women aged 15–49 years, currently married or in union, and in need of contraception (unweighted).

**Primary and secondary outcome measures:**

Demand for family planning satisfied by modern methods (mDFPS) and wealth-related inequalities in mDFPS, assessed using the slope index of inequality (SII) and the concentration index (CIX).

**Results:**

In both income groups, at the province level, higher mean women’s schooling and greater proportion of employed women were positively associated with mDFPS coverage. In contrast, higher male-to-female educational attainment ratios were inversely associated with mDFPS. Provinces with higher means of women’s schooling also showed lower wealth-related inequalities in mDFPS. At the individual level, women residing in provinces with higher male-to-female educational attainment ratios were found to have lower odds of mDFPS, regardless of the income group. Additionally, the factors influencing individual women’s mDFPS varied depending on the income level of the country’s provinces.

**Conclusion:**

Women’s empowerment and gender equality in education at the province level significantly influence family planning outcomes. Targeted interventions that address each region’s specific educational, economic and demographic contexts are crucial for improving coverage and reducing disparities in family planning services.

**STRENGTHS AND LIMITATIONS OF THIS STUDY**

- This study addresses a gap in the literature by examining how province-level contextual factors influence family planning outcomes across multiple countries.
- The study used standardised and provincially representative Demographic and Health Surveys data from 46 low- and middle-income countries.
- A multilevel modelling approach accounted for variance at the individual, province and country levels.
- Part of the analysis is ecological, limiting causal inference due to potential ecological bias, but findings are consistent with individual-level patterns, reducing related concerns.
- Important variables such as family planning availability and healthcare system characteristics were not included in the models.

## Introduction

 Women have the basic right to determine the number and spacing of their children. Guaranteeing the access of women and adolescents to family planning information and safe and effective contraceptive methods of their choice is key to promoting health and well-being.^1^ Contraceptive use prevents unintended pregnancies and reduces the likelihood of closely spaced births, decreasing the occurrence of high-risk pregnancies, poor perinatal outcomes and maternal and child mortality.^2^,^4^ Additionally, it enables women to pursue higher education, increases their career opportunities and promotes financial independence.^5 6^

Global attempts have been made to encourage access to family planning services. These are exemplified by initiatives such as the United Nations Sustainable Development Goals (SDGs), in which Target 3.7 aims to ensure, by 2030, universal access to sexual and reproductive healthcare services, including family planning, information and education and the integration of reproductive health into national strategies and programmes.^7^ The use of modern contraceptive methods is one of the most effective means to prevent unintended pregnancies.^8^ However, access to these methods varies significantly across regions. While short-term methods such as pills and condoms show relatively high and consistent availability—exceeding over 75% in all regions—access to long-term methods remains uneven. Female and male sterilisation are more accessible in Asia, intrauterine devices in Eastern Europe and Central Asia and the Middle East and North Africa, and injectables and implants in sub-Saharan Africa.^9^ In 2019, the global modern contraceptive prevalence rate stood at 47.7%, varying from 23.6% in sub-Saharan Africa to 64.7% in Southeast Asia, East Asia and Oceania.^10^

The proportion of women of reproductive age whose demand for family planning is satisfied with modern methods (mDFPS) is a valuable indicator in assessing overall levels of coverage for family planning programmes and services.^11^ Globally, mDFPS increased from 66.9% in 1990 to 77.4% in 2021,^12^ with important reductions in wealth-related inequalities in countries of different income levels.^13^ Despite these improvements, low levels of mDFPS are still observed in low-income countries. In 2019, mDFPS was 79.9% in high-income countries, contrasting with 52.6% in low-income nations.^14^ Furthermore, the progress in increasing modern contraceptive use varies across countries,^10 15^ and significant within-country disparities persist concerning economic status, education, age and area of residence.^16^

Understanding the drivers of mDFPS coverage and inequalities is vital to support the design and implementation of targeted family planning interventions and programmes that are more effective and could contribute to achieving SDG Target 3.7. Previous studies have assessed whether cultural, social, socioeconomic and geographical contexts affect family planning. Most of the evidence is derived from country-specific or multicountry analyses focusing on the local community context, such as using the primary sampling units from Demographic and Health Surveys (DHS) as a proxy for these communities. Multicountry studies were conducted with countries from Africa,^17^,^27^ Latin America and the Caribbean,^28^ and low- and middle-income countries (LMICs) globally.^29 30^ Still, no contextual factors were investigated at the provincial level in multicountry studies. Given the heterogeneity of provinces and the need for solutions tailored to their specific needs, we aimed to assess (1) the extent to which contextual factors are associated with the coverage and wealth-related inequalities in mDFPS across provinces in LMICs and (2) whether these factors influence mDFPS at women’s level.

## Methods

### Data source

This observational study used data from the DHS in LMICs. The DHS is a series of nationally representative surveys that provide comprehensive information on various health-related topics. Employing a multistage cluster sampling design, the DHS sample is representative at the national level and at the first subnational administrative level, namely regions, provinces, zones, departments or states, referred to as ‘provinces’ in this study. 46 countries were included in the analyses, consisting of surveys carried out since 2010 and with complete data for all variables. Only the most recent survey was considered when multiple surveys were available for a country.

### Outcomes

mDFPS was defined as women aged 15–49 years currently married or in union in need of contraception who were currently using, or whose sexual partner was currently using, at least one modern contraceptive method. Women in need of contraception are those who are fecund but want to either stop or delay childbearing and those who are pregnant or experiencing postpartum amenorrhoea (no menstrual period since their last live birth in the previous 2 years) with the current pregnancy or last birth being mistimed or unwanted.^31^ Following the proposal of Hubacher and Trussel,^32^ modern methods of contraception are defined as technological products or medical procedures that prevent natural reproduction and include the following: sterilisation (male and female), intrauterine devices and systems, subdermal implants, oral contraceptives, condoms (male and female), injectables, emergency contraceptive pills, patches, diaphragms and cervical caps, spermicidal agents (gels, foams, creams, suppositories, etc), vaginal rings and sponge. Following this definition, this study did not consider lactational amenorrhoea and fertility awareness as modern methods. mDFPS was computed at the individual level by identifying women whose family planning needs were satisfied with modern methods. Then, the proportion of women with satisfied needs was aggregated at the province level for use in ecological analyses.

Two wealth-related inequality indices for mDFPS were estimated at the province level: the slope index of inequality (SII) and the concentration index (CIX). The SII was estimated through a logistic regression model and represents the absolute difference in the predicted value of the outcome between the highest and lowest quintiles of the wealth index within each province. Parallel to the Gini index, the CIX measures relative inequality by calculating the area between the concentration curve and the ideal line of perfect equality. It was derived by plotting the cumulative fraction of the sample ranked by household wealth index against the cumulative fraction of the outcome. Both indices range from −1 to +1 but were multiplied by 100 to facilitate interpretation. Positive values indicate higher mDFPS among the wealthy, reflecting a pro-rich pattern, while negative values indicate higher mDFPS among the poor, reflecting a pro-poor pattern.

### Predictors

The province-level predictor variables, selected based on previous findings in literature and theoretical knowledge, are summarised in online supplemental table 1. They represent domains of standards and practices related to gender roles and dynamics, such as women’s empowerment (women’s education, employment and two domains of a survey-based women’s empowerment index—the SWPER Global), gender disparity (male-to-female educational attainment ratio) and social practices (women’s early marriage and adolescent childbearing). We also assessed variables representing the urbanisation level, women’s age composition and absolute income. At the country level, we evaluated the national gross domestic product (GDP) per capita based on purchasing power parity (current international dollars), obtained from the World Bank database^33^ and expressed on a logarithmic scale (lnGDP). For each country included in the analysis, we used the GDP per capita from the year when the survey was conducted.

The SWPER Global index is estimated by principal component analyses using 14 DHS questions. It was derived using data at the individual level from 62 LMICs and represents three women’s empowerment domains: (1) attitudes to violence, as a proxy for women’s incorporation of gender norms-related acceptability of violence; (2) decision-making, the level of women’s participation in household decisions; and (3) social independence, mainly composed of conditions that enable women to achieve their goals (schooling attainment, access to information, age at pivotal life events and spousal asset differentials). Further details are provided elsewhere.^34^ The scores for each domain were classified into three tertiles, creating groups of low, medium and high levels of empowerment. We evaluated the prevalence of high empowerment in the attitude towards violence and decision-making domains by grouping the first two tertiles (low and medium). The social independence domain was excluded from the analysis due to collinearity with women’s education (r=0.858, p<0.001) and early marriage (r=−0.803, p<0.001). While we recognise that the social independence domain encompasses broader aspects of empowerment, we opted to use those variables as proxies.

To generate the median annual household absolute income of each province, the absolute income distribution for each country was computed based on the share of the consumption from each of the country’s GDP, obtained from the World Bank,^33^ and the Gini coefficient, sourced from the Standardised World Income Inequality Database,^35^ following the methodology proposed by Fink *et al*.^36^ The average absolute income in US dollars was determined for N quantiles, with N matching the total number of clusters in the country. Subsequently, households in the survey sample were ranked based on N quantiles of the wealth index score, and each household within a wealth index quantile was allocated the dollar value corresponding to the same quantile of the absolute income distribution. The median annual household absolute income of a province corresponds to the value at the 50th percentile of the assigned household absolute income within the province.

### Statistical analysis

First, an ecological analysis at the province level was performed. Median and IQR were employed for descriptive analysis. Pearson’s correlation coefficients were calculated to assess the correlation among variables. Pairs with a correlation coefficient greater than 0.8 were considered collinear, and collinear variables were removed accordingly. The association of contextual factors with coverage and wealth-related inequalities in mDFPS at the province level was evaluated through crude and adjusted multilevel linear regression. This analysis followed a two-level hierarchical structure, with level 2 representing countries and level 1 corresponding to provinces within those countries. Subsequently, crude and adjusted multilevel logistic regression analyses were conducted to evaluate the contextual factors associated with individual women’s mDFPS. To account for individual-level effects, corresponding individual measures were included in the model, forming a hierarchical structure with three levels: level 3 denoted countries, level 2 provinces and level 1 individuals. Adjusted analyses included all predictor variables in their respective models. Both multilevel linear and logistic regression models employed random intercept models. Additionally, we computed the total variance attributed to country and province levels. The analyses were stratified based on the World Bank’s income classifications, distinguishing between (1) low-income countries (LICs) and (2) middle-income countries (MICs), with the latter comprising both lower-middle and upper-middle-income countries. All analyses considered the multistage survey design and incorporated sampling weights. For the analysis of mDFPS at the individual level, weights were adjusted using the population size of women aged 15–49 in each country in the median year of the surveys (LICs: 2016, MICs: 2018). The weights were further rescaled through linear normalisation. All analyses were conducted using Stata V.18.0.

## Results

Complete data were available for 46 countries, 621 provinces and 302 493 women aged 15–49 years, currently married or in union, and in need of contraception (unweighted). Table 1 describes the surveys, countries and provinces analysed in this study. The surveys covered the period from 2011 to 2022, and the number of provinces per country ranged from 3 (Comoros and Malawi) to 47 (Kenya). The countries’ median lnGDP per capita ranged from 6.6 in the Democratic Republic of the Congo (equivalent to US$732) to 9.6 in Gabon (equivalent to US$15 092). The provinces’ mDFPS coverage showed considerable variation, with a median of 55.2% and ranging from 0.4% to 93.4% across all provinces. Additionally, regarding wealth-related inequalities, the provinces showed a median SII of 8.5 percentage points, ranging from −40.8 (favouring the poor) to 89.6 (favouring the rich) and a median CIX of 2.9, ranging from −20.4 (favouring the poor) to 57.8 (favouring the rich). Online supplemental table 2 lists countries included in the analysis and their provinces’ median mDFPS, SII and CIX.

**Table 1:** Survey and country characteristics and descriptive analysis at province level

Variable	Median	IQR	Min	Max
Country-level characteristics (n=46)				
Survey and country characteristics				
Survey year	2017	–	2011	2022
Number of provinces per country	15	–	3	47
Country lnGDP per capita, PPP	8.2	1	6.6	9.6
Province-level characteristics (n=621)				
Family planning				
mDFPS coverage (%)	55.2	37	0.4	93.4
Wealth-related inequalities				
SII mDFPS	8.5	23	−40.8	89.6
CIX mDFPS	2.9	10	−20.4	57.8
Urbanisation and population composition				
Urban population (%)	27.8	33	1.3	100.0
Mean women’s age (years)	28.9	2	25.6	33.6
Median absolute income (US$)	7786	7989	1151	52 900
Women’s empowerment				
Mean women’s schooling (years)	6.7	5	0.2	14.5
Women currently employed (%)	52.0	28	5.1	95.8
High SWPER attitude to violence (%)	62.0	32	5.0	98.8
High SWPER decision-making (%)	64.1	29	0.0	97.9
Gender disparity				
M/F educational attainment ratio	1.3	1	0.2	14.5
Social practices				
Early marriage (%)	23.5	24	0.4	83.9
Adolescent childbearing (%)	16.0	16	0.0	59.6

High SWPER scores reflect higher women’s empowerment.

CIX, concentration index; lnGDP, natural logarithm of gross domestic product; mDFPS, demand for family planning satisfied by modern methods; M/F, male-to-female; PPP, purchasing power parity; SII, slope index of inequality; SWPER, survey-based women’s empowerment index.

Online supplemental table 3 presents the correlation matrix for the province-level contextual factors and outcomes. Mean women’s age, median absolute income, mean women’s schooling and proportion of women with high empowerment in all three SWPER domains were positively associated with provincial mDFPS and inversely related to absolute and relative inequalities. Conversely, the male-to-female educational attainment ratio, proportion of early marriage and proportion of adolescent childbearing were inversely associated with provincial mDFPS and directly related to wealth-related inequality measures. In addition, the urban population proportion showed an inverse association with absolute inequality.

**Figure 1:**
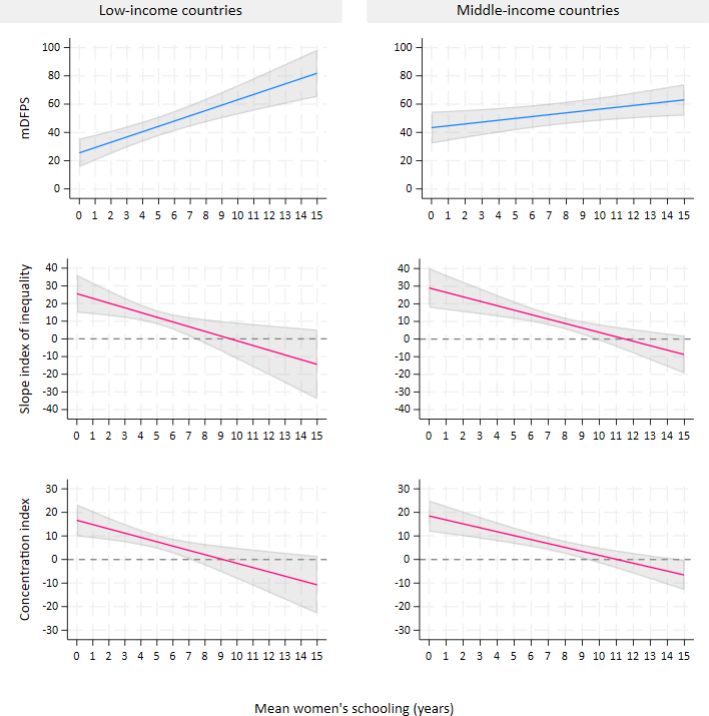
Adjusted predictions of mDFPS, slope index of inequality and concentration index according to mean women’s schooling, by low- and middle-income countries (ecological analysis at province level). The lines represent the average adjusted predictions of the outcomes for mean schooling ranging from 0 to 15 years, derived from a multilevel linear regression model. The grey area indicates the corresponding confidence interval. mDFPS, demand for family planning satisfied by modern methods.

Table 2 shows the association between contextual factors and the coverage and wealth-related inequalities in mDFPS across provinces in LICs. Adjusted analyses indicated that relative inequality tended to be higher in provinces with a larger urban population proportion (β=0.09, 95% CI 0.01 to 0.17) and higher mean women’s age (β=2.05, 95% CI 0.40 to 3.71). Concerning women’s empowerment, a consistent positive association was observed between mean women’s schooling and the outcomes, with associations strengthening after adjustment (figure 1). A strong positive relationship with mDFPS became evident, indicating that, on average, each additional year of mean schooling corresponded to a 3.76 percentage point increase in mDFPS (95% CI 2.25 to 5.27). Furthermore, mean women’s schooling was associated with a reduction of −2.67 in SII (95% CI −4.62 to −0.72) and −1.83 in CIX (95% CI −3.03 to −0.63). In adjusted models, the proportion of currently employed women was positively associated with mDFPS and negatively associated with relative inequality, while the male-to-female educational attainment ratio showed an inverse association with mDFPS in both crude and adjusted analysis. Including contextual variables in the adjusted models reduced outcome variance at both the country and province levels, when compared with their respective null models.

**Table 2:** Association of contextual factors with coverage and wealth-related inequality measures in mDFPS in low-income countries (ecological analysis at province level). N=204 provinces

Variables	mDFPS (%)					SII mDFPS					CIX mDFPS				
	Null	Crude		Adjusted		Null	Crude		Adjusted		Null	Crude		Adjusted	
		β (95% CI)	P value	β (95% CI)	P value		β (95% CI)	P value	β (95% CI)	P value		β (95% CI)	P value	β (95% CI)	P value
Country-level															
LnGDP per capita, PPP		11.99 (−9.72 to 33.70)	0.28	11.56 (−5.75 to 28.86)	0.19		−5.02 (−17.25 to 7.22)	0.42	−3.17 (−14.17 to 7.83)	0.57		−5.22 (−13.75 to 3.31)	0.23	−5.17 (−13.01 to 2.68)	0.20
Province-level															
Urban population (%)		0.06 (0.01 to 0.12)	0.03	−0.06 (−0.16 to 0.04)	0.25		−0.05 (−0.13 to 0.03)	0.18	0.07 (−0.08 to 0.21)	0.35		−0.04 (−0.08 to 0.01)	0.06	0.09 (0.01 to 0.17)	0.03
Mean women’s age (years)		0.17 (−2.03 to 2.37)	0.88	−0.29 (−2.47 to 1.90)	0.80		−2.68 (−5.68 to 0.31)	0.08	−1.97 (−4.99 to 1.04)	0.20		1.19 (−0.50 to 2.87)	0.17	2.05 (0.40 to 3.71)	0.02
Median absolute income (in US$1000)		0.50 (0.10 to 0.89)	0.01	−0.17 (−0.82 to 0.48)	0.61		−0.45 (−0.97 to 0.08)	0.10	0.25 (−0.68 to 1.17)	0.60		−0.38 (−0.67 to −0.08)	0.01	−0.01 (−0.51 to 0.49)	0.97
Mean women’s schooling (years)		2.78 (1.87 to 3.68)	<0.01	3.76 (2.25 to 5.27)	<0.01		−2.46 (−3.63 to −1.29)	<0.01	−2.67 (−4.62 to −0.72)	<0.01		−1.67 (−2.35 to −0.99)	<0.01	−1.83 (−3.03 to −0.63)	<0.01
Women currently employed (%)		0.20 (0.07 to 0.33)	<0.01	0.18 (0.06 to 0.31)	<0.01		−0.10 (−0.29 to 0.10)	0.32	−0.19 (−0.39 to 0.01)	0.05		−0.08 (−0.19 to 0.04)	0.18	−0.15 (−0.26 to −0.04)	<0.01
High SWPER attitude to violence (%)		−0.02 (−0.13 to 0.09)	0.68	−0.02 (−0.14 to 0.11)	0.81		−0.16 (−0.30 to −0.01)	0.03	−0.08 (−0.25 to 0.08)	0.31		−0.07 (−0.15 to 0.02)	0.11	−0.01 (−0.11 to 0.08)	0.80
High SWPER decision-making (%)		0.17 (0.03 to 0.32)	0.02	0.08 (−0.07 to 0.23)	0.28		−0.17 (−0.33 to −0.02)	0.03	0.06 (−0.12 to 0.24)	0.52		−0.12 (−0.22 to −0.03)	0.01	−0.01 (−0.12 to 0.10)	0.84
M/F educational attainment ratio		−1.28 (−2.06 to −0.51)	<0.01	−0.90 (−1.66 to −0.14)	0.02		0.35 (−0.82 to 1.51)	0.56	−0.51 (−1.71 to 0.69)	0.41		0.28 (−0.38 to 0.94)	0.40	−0.21 (−0.87 to 0.44)	0.53
Early marriage (%)		−0.19 (−0.31 to −0.07)	<0.01	0.08 (−0.11 to 0.26)	0.44		0.37 (0.22 to 0.52)	<0.01	0.21 (−0.05 to 0.47)	0.11		0.22 (0.14 to 0.31)	<0.01	0.07 (−0.08 to 0.21)	0.35
Adolescent childbearing (%)		−0.14 (−0.33 to 0.04)	0.13	0.05 (−0.19 to 0.28)	0.71		0.49 (0.25 to 0.73)	<0.01	0.08 (−0.26 to 0.42)	0.65		0.35 (0.21 to 0.48)	<0.01	0.18 (−0.01 to 0.38)	0.06
Variance															
Between-countries	406.2			227.8		109.5			54.2		59.5			36.8	
Between-provinces	107.1			83.7		199.5			172.9		60.4			48.1	

Multilevel linear regression. Beta coefficients represent the change in the outcome associated with a 1-unit increase in the predictor. Positive values indicate a direct association, while negative values indicate an inverse association. High SWPER scores reflect higher women’s empowerment.

CIX, concentration index; lnGDP, natural logarithm of gross domestic product; mDFPS, demand for family planning satisfied by modern methods; M/F, male-to-female; PPP, purchasing power parity; SII, slope index of inequality; SWPER, survey-based women’s empowerment index.

Table 3 displays the association between contextual factors and the coverage and wealth-related inequalities in mDFPS in MICs. After adjustment, the association between urbanisation and the outcomes disappeared, while mean women’s age remained positively associated with mDFPS (β=1.97, 95% CI 0.65 to 3.28) and inversely related to absolute inequality (β=−2.76, 95% CI −4.57 to −0.94), although the association was less pronounced. As observed in LICs, higher mean women’s schooling was associated with increased mDFPS and reduced absolute and relative inequalities (figure 1), in both crude and adjusted analysis. The proportion of currently employed women was positively associated with mDFPS and inversely associated with absolute inequality. Additionally, provinces with greater percentages of women with high empowerment in the SWPER decision-making domain tended to have lower absolute inequality. After adjustment, the male-to-female educational attainment ratio showed a strong inverse association with mDFPS (β=−3.56, 95% CI −5.81 to −1.32) and a direct association with relative inequality (β=1.99, 95% CI 0.42 to 3.57). In addition, absolute inequality was slightly higher in provinces with a higher proportion of adolescent childbearing. The inclusion of contextual variables in the adjusted models reduced the variance of outcomes at both the country and province levels, compared with their respective null models.

**Table 3:** Association of contextual factors with coverage and wealth-related inequality measures in mDFPS in middle-income countries (ecological analysis at province level). N=417 provinces

Variables	mDFPS (%)					SII mDFPS					CIX mDFPS				
	Null	Crude		Adjusted		Null	Crude		Adjusted		Null	Crude		Adjusted	
		β (95% CI)	P value	β (95% CI)	P value		β (95% CI)	P value	β (95% CI)	P value		β (95% CI)	P value	β (95% CI)	P value
Country-level															
LnGDP per capita, PPP		−4.07 (−19.74 to 11.59)	0.61	−9.78 (−25.57 to 6.02)	0.23		−5.52 (−14.47 to 3.42)	0.23	4.19 (−3.16 to 11.55)	0.26		−0.73 (−7.27 to 5.81)	0.83	4.48 (−1.25 to 10.21)	0.13
Province-level															
Urban population (%)		0.07 (0.02 to 0.13)	<0.01	−0.06 (−0.13 to 0.01)	0.06		−0.15 (−0.22 to −0.09)	<0.01	−0.07 (−0.16 to 0.03)	0.17		−0.10 (−0.13 to −0.06)	<0.01	−0.01 (−0.06 to 0.04)	0.57
Mean women’s age (years)		4.90 (3.57 to 6.22)	<0.01	1.97 (0.65 to 3.28)	<0.01		−5.37 (−7.01 to −3.73)	<0.01	−2.76 (−4.57 to −0.94)	<0.01		−1.85 (−2.82 to −0.87)	<0.01	−0.21 (−1.18 to 0.75)	0.66
Median absolute income (in US$1000)		0.60 (0.41 to 0.79)	<0.01	0.28 (0.01 to 0.57)	0.05		−0.53 (−0.78 to −0.28)	<0.01	0.34 (−0.02 to 0.70)	0.06		−0.36 (−0.49 to −0.23)	<0.01	0.09 (−0.11 to 0.28)	0.38
Mean women’s schooling (years)		2.90 (2.24 to 3.57)	<0.01	1.31 (0.24 to 2.38)	0.02		−3.71 (−4.59 to −2.83)	<0.01	−2.52 (−3.92 to −1.12)	<0.01		−2.45 (−2.91 to −1.99)	<0.01	−1.67 (−2.46 to −0.88)	<0.01
Women currently employed (%)		0.48 (0.38 to 0.57)	<0.01	0.32 (0.22 to 0.42)	<0.01		−0.26 (−0.41 to −0.11)	<0.01	−0.20 (−0.33 to −0.06)	<0.01		−0.09 (−0.17 to −0.01)	0.04	−0.05 (−0.12 to 0.02)	0.17
High SWPER attitude to violence (%)		0.12 (0.04 to 0.20)	<0.01	0.00 (−0.07 to 0.08)	0.93		−0.17 (−0.27 to −0.07)	<0.01	−0.03 (−0.13 to 0.07)	0.54		−0.09 (−0.14 to −0.04)	<0.01	0.00 (−0.05 to 0.06)	0.88
High SWPER decision-making (%)		0.12 (0.02 to 0.22)	0.02	−0.04 (−0.13 to 0.05)	0.41		−0.34 (−0.45 to −0.22)	<0.01	−0.13 (−0.26 to −0.01)	0.04		−0.17 (−0.23 to −0.11)	<0.01	−0.02 (−0.09 to 0.05)	0.61
M/F educational attainment ratio		−7.75 (−9.97 to −5.52)	<0.01	−3.56 (−5.81 to −1.32)	<0.01		5.91 (2.89 to 8.93)	<0.01	0.36 (−2.74 to 3.45)	0.82		4.98 (3.43 to 6.52)	<0.01	1.99 (0.42 to 3.57)	0.01
Early marriage (%)		−0.34 (−0.44 to −0.24)	<0.01	−0.08 (−0.23 to 0.06)	0.25		0.41 (0.28 to 0.55)	<0.01	−0.11 (−0.30 to 0.08)	0.26		0.31 (0.24 to 0.38)	<0.01	0.06 (−0.04 to 0.17)	0.22
Adolescent childbearing (%)		−0.33 (−0.50 to −0.17)	<0.01	0.10 (−0.09 to 0.29)	0.30		0.65 (0.45 to 0.86)	<0.01	0.30 (0.03 to 0.56)	0.03		0.41 (0.29 to 0.52)	<0.01	0.08 (−0.06 to 0.23)	0.25
Variance															
Between-countries	407.0			401.5		123.3			54.7		67.2			45.6	
Between-provinces	133.7			90.3		216.8			174.2		57.1			42.4	

Multilevel linear regression. Beta coefficients represent the change in the outcome associated with a 1-unit increase in the predictor. Positive values indicate a direct association, while negative values indicate an inverse association. High SWPER scores reflect higher women’s empowerment.

CIX, concentration index; lnGDP, natural logarithm of gross domestic product; mDFPS, demand for family planning satisfied by modern methods; M/F, male-to-female; PPP, purchasing power parity; SII, slope index of inequality; SWPER, survey-based women’s empowerment index.

Table 4 presents the crude and adjusted association of contextual and individual factors with individual women’s mDFPS by World Bank income groups. After adjusting for country-level and individual-level factors, we found a direct association between mean women’s schooling at the province level and mDFPS among women from LICs: a 1-year increase in mean province-level schooling led to a 14% rise in the odds of mDFPS (OR=1.14, 95% CI 1.04 to 1.26). Adjusted analyses showed that mean women’s age (OR=1.10, 95% CI 1.01 to 1.21) and median absolute income (OR=1.01, 95% CI 1.00 to 1.02) at the province level were positively associated with the odds of mDFPS in MICs. Moreover, women living in provinces with higher male-to-female educational attainment ratio were found to have lower odds of having their demand for family planning satisfied by modern contraceptive methods in both LICs (OR=0.94, 95% CI 0.89 to 0.99) and MICs (OR=0.80, 95% CI 0.67 to 0.97). When comparing the null and adjusted models, it was observed that the between-country variance in mDFPS decreased by 39% in LICs, while the between-provinces variance changed by 8%. Conversely, in MICs, no change was observed in the between-country variance, while the between-provinces variance decreased by 40%.

**Table 4:** Association of contextual and individual factors with women’s individual mDFPS in low- and middle-income countries. N=302 493 women

Variables	Low-income countries (N=95 437)					Middle-income countries (N=207 056)				
	Null	Crude		Adjusted		Null	Crude		Adjusted	
		OR (95% CI)	P value	OR (95% CI)	P value		OR (95% CI)	P value	OR (95% CI)	P value
Country-level										
LnGDP per capita, PPP		1.81 (0.74 to 4.44)	0.19	1.98 (0.83 to 4.72)	0.12		0.79 (0.31 to 2.04)	0.63	0.62 (0.23 to 1.68)	0.35
Province-level										
Urban population (%)		1.00 (1.00 to 1.01)	<0.01	1.00 (0.99 to 1.00)	0.65		1.00 (1.00 to 1.01)	0.02	1.00 (1.00 to 1.01)	0.74
Mean women’s age (years)		0.96 (0.84 to 1.10)	0.59	0.96 (0.84 to 1.11)	0.58		1.20 (1.09 to 1.32)	<0.01	1.10 (1.01 to 1.21)	0.03
Median absolute income (in US$1000)		1.02 (1.01 to 1.04)	<0.01	0.98 (0.95 to 1.01)	0.18		1.02 (1.01 to 1.04)	<0.01	1.01 (1.00 to 1.02)	0.02
Mean women’s schooling (years)		1.12 (1.06 to 1.17)	<0.01	1.14 (1.04 to 1.26)	<0.01		1.11 (1.04 to 1.17)	<0.01	1.03 (0.96 to 1.11)	0.36
Women currently employed (%)		1.00 (1.00 to 1.01)	0.25	1.01 (1.00 to 1.02)	0.08		1.01 (1.00 to 1.02)	<0.01	1.01 (1.00 to 1.01)	0.07
High SWPER attitude to violence (%)		1.00 (1.00 to 1.01)	0.51	1.00 (0.99 to 1.01)	0.76		1.00 (0.99 to 1.01)	0.61	1.00 (0.99 to 1.00)	0.24
High SWPER decision-making (%)		1.01 (1.00 to 1.02)	0.01	1.00 (1.00 to 1.01)	0.34		1.01 (1.00 to 1.01)	0.05	1.00 (0.99 to 1.00)	0.49
M/F educational attainment ratio		0.93 (0.88 to 0.98)	<0.01	0.94 (0.89 to 0.99)	0.02		0.73 (0.60 to 0.88)	<0.01	0.80 (0.67 to 0.97)	0.02
Early marriage (%)		0.99 (0.98 to 1.00)	0.01	1.00 (0.99 to 1.01)	0.49		0.99 (0.98 to 1.00)	<0.01	1.00 (0.99 to 1.01)	0.79
Adolescent childbearing (%)		0.99 (0.98 to 1.00)	0.04	1.00 (0.99 to 1.01)	0.98		0.98 (0.97 to 1.00)	0.01	1.00 (0.98 to 1.01)	0.68
Individual-level										
Urban area		1.26 (1.06 to 1.51)	0.01	1.05 (0.92 to 1.21)	0.47		1.06 (0.86 to 1.32)	0.57	0.96 (0.87 to 1.06)	0.42
Age (years)		1.00 (0.99 to 1.01)	0.74	1.00 (0.99 to 1.01)	0.83		1.01 (0.99 to 1.03)	0.38	1.01 (0.99 to 1.03)	0.57
Wealth index (quintiles)*		1.10 (1.05 to 1.16)	<0.01	1.07 (1.03 to 1.12)	<0.01		1.05 (0.97 to 1.13)	0.22	1.07 (1.02 to 1.11)	<0.01
Schooling (completed years)		1.04 (1.02 to 1.06)	<0.01	1.03 (1.01 to 1.04)	<0.01		1.00 (0.97 to 1.03)	0.95	1.00 (0.97 to 1.02)	0.72
Currently employed		1.30 (1.21 to 1.41)	<0.01	1.30 (1.23 to 1.38)	<0.01		1.29 (1.14 to 1.46)	<0.01	1.24 (1.15 to 1.33)	<0.01
High SWPER attitude to violence domain		1.05 (0.98 to 1.13)	0.19	0.99 (0.94 to 1.03)	0.54		1.01 (0.94 to 1.09)	0.77	0.99 (0.95 to 1.03)	0.62
High SWPER decision-making domain		1.18 (1.08 to 1.28)	<0.01	1.12 (1.06 to 1.19)	<0.01		1.14 (1.05 to 1.24)	<0.01	1.10 (1.05 to 1.15)	<0.01
First married/in union before age 18		0.99 (0.92 to 1.08)	0.87	0.94 (0.88 to 1.00)	0.04		1.10 (0.97 to 1.23)	0.12	0.99 (0.94 to 1.05)	0.77
First birth before age 20		1.14 (1.04 to 1.24)	<0.01	1.27 (1.18 to 1.38)	<0.01		1.20 (1.07 to 1.34)	<0.01	1.27 (1.16 to 1.39)	<0.01
Variance										
Between-countries	0.89			0.54		0.99			0.99	
Between-provinces	0.13			0.12		0.10			0.06	
Between-individuals	3.29			3.29		3.29			3.29	

High SWPER scores reflect higher women’s empowerment.

* Wealth index quintiles within province. Multilevel logistic regression.

lnGDP, natural logarithm of gross domestic product; M/F, male-to-female; PPP, purchasing power parity; SWPER, survey-based women’s empowerment index.

## Discussion

Our ecological analysis revealed that, in LICs and MICs, provinces with higher mean women’s schooling and a greater proportion of currently employed women tended to have higher mDFPS coverage. In contrast, provinces with higher male-to-female educational attainment ratios were associated with lower mDFPS. Regarding inequalities in mDFPS, provinces with higher mean women’s schooling exhibited lower wealth-related inequalities in mDFPS, irrespective of the countries’ income group. Our analysis of mDFPS at the individual level showed an inverse association between the provincial male-to-female educational attainment ratio and women’s mDFPS across both income groups. Moreover, factors influencing individual women’s mDFPS varied depending on the income level of the country’s provinces.

We identified substantial variation in mDFPS across provinces, which aligns with findings from other studies.^37^,^43^ This provincial variability exceeds the observed variability across countries in previous research,^1013^,^15 43^ emphasising the need to disaggregate data at subnational levels to uncover differences that may be masked in national-level analyses. Moreover, considerable variability in wealth-related inequalities in mDFPS was observed, ranging from pro-poor to pro-rich patterns, as documented in other country-level studies.^43 44^

The findings presented in this study reflect the critical role of women’s empowerment in reproductive health. Empowerment is a multidimensional concept that encompasses different aspects, such as education and economic participation, both of which contribute to improved reproductive health outcomes. Education, for instance, enhances women’s knowledge of sexual and reproductive health, enabling them to make informed contraceptive choices.^45^ Economic participation promotes financial independence, broadening access to healthcare resources.^46^ In regions where women are more empowered, positive gender norms tend to develop,^47 48^ supporting better reproductive health practices and strengthening healthcare infrastructure.^49^ We observed that higher levels of female education within provinces are associated with better coverage and reduced wealth-related inequalities in mDFPS. Specifically, in LICs, once the mean women’s education reached 7–8 years, and in MICs, 9–10 years, wealth-related inequalities in mDFPS disappeared. These results suggest that increasing women’s education helps to overcome socioeconomic barriers, making family planning services more universally accessible. Additionally, we found that more employed women are positively associated with mDFPS coverage in provinces. Given the strong correlation between female education and empowerment found in our study, education stands out as a key aspect of empowerment and a critical area for investment, offering a strategic approach to improving reproductive health outcomes.

We further demonstrated how gender disparity in education at the provincial level negatively impacts both coverage and individual mDFPS. Gender equity can promote women’s reproductive rights by providing access to comprehensive health services, supporting informed choices about their bodies and allowing full control over their reproductive lives.^50^ Although global progress has been made in reducing gender inequality in education, it remains unevenly distributed.^51 52^ For instance, while gender parity in school enrolment has been achieved in primary and lower secondary education globally, substantial disparities persist in sub-Saharan Africa, where boys continue to have a considerable advantage at all educational levels.^52^ To enhance women’s health outcomes, efforts are still needed to close the gender gap and achieve educational parity.

It is important to recognise the potential bidirectionality of these relationships. While women’s empowerment and gender equality can improve family planning, the reverse is also true: family planning can positively impact educational and career opportunities, as well as contribute to reducing gender inequalities, by enabling girls and young women to avoid unintended pregnancies and manage their reproductive health.^5 6^ Thus, the relationship between family planning and factors such as women’s empowerment and gender equality is interdependent and can be viewed as a cyclical process. Our results highlighted key provincial contextual drivers of family planning, providing guidance for the development of targeted interventions that strengthen and sustain this process.

Our findings also revealed that factors influencing mDFPS may vary depending on the income level of the country’s provinces. In LICs, higher levels of women’s education at the province level were associated with increased individual mDFPS, suggesting that education may be central in overcoming barriers to reproductive health services in these settings. In contrast, in MICs, higher household incomes and an older average age of women at the province level were associated with greater individual mDFPS. This trend is likely due to the superior healthcare quality typically found in wealthier regions.^53^ Furthermore, the older average age of women in these provinces may reflect a more advanced stage in the demographic transition, in which societies experience changes in attitudes toward family planning.^54^ It is also important to note that, in MICs, the average level of education is already relatively high compared with LICs, which may explain why the impact of education on mDFPS is less pronounced in these settings. Considering these variations, interventions to address family planning needs must be designed to the specific income level of the country where each province is located.

When interpreting the results of this study, some limitations must be considered. Part of our results is based on ecological analysis, which is subject to ecological bias and limits our ability to infer causality. Nevertheless, the observed associations are consistent with individual-level patterns, helping to reduce concerns about ecological fallacy. In addition, our analysis focused exclusively on currently married or in-union women. This approach allowed us to evaluate a larger number of countries and use the SWPER global index, which is specifically calculated for married or in-union women. However, this focus may limit the generalisability of our findings. The year of the surveys may also be a limitation, as we compared more recent surveys with older ones, and the effects of variables may differ across different temporal contexts. Furthermore, important contextual factors—such as family planning availability, cultural norms and the development of healthcare and public health systems, particularly at the provincial level—were not included in the analysis, despite their potential influence on outcomes. Validated national-level indices of inequality, such as the United Nations Gender Inequality Index, provide valuable information but were not incorporated because such indices are not available at the provincial level, which is the focus of this study.

Regardless of these limitations, our study offers notable strengths. First, this study addresses a gap in the literature by analysing province-level contextual factors associated with family planning outcomes across multiple countries. Although analyses at more proximate levels may yield more accurate findings, focusing on provinces captures broader contextual factors important for region-specific strategies. Previous multicountry studies have used community-level data, relying on DHS primary sample units as proxies for communities and have shown the positive impact of community education^17 18 20 22 24 29^ and the role of gender inequality in education^30^ on family planning outcomes. Likewise, a country-level study focusing on Latin American countries observed an inverse association between gender inequality and mDFPS.^28^ Second, our study encompasses a diverse range of provinces from LMICs, using comparable standardised surveys and representative data at the province level, allowing for robust cross-contextual comparisons.

In conclusion, our study emphasises the importance of women’s empowerment and gender equality in education in achieving family planning outcomes. It draws attention to the potential of provinces as focal points for interventions. Given that provinces often have administrative and policymaking autonomy, policies and programmes should be developed to address the specific educational, economic and demographic contexts of each region. To optimise their effectiveness in achieving family planning goals, it is essential to tailor interventions based on the income levels of the country’s provinces.

## Supplementary material

10.1136/bmjopen-2025-098980 online supplemental file 1

## Data Availability

Data are available in a public, open access repository.
