# Supplementary material for: Influence of subnational contextual factors on demand for family planning satisfied by modern methods: a multilevel approach in 46 surveys from low- and middle-income countries
Source: BMJ Open. 2025 Nov 23;15(11):e098980. doi: 10.1136/bmjopen-2025-098980 (PMC12645598; doi:10.1136/bmjopen-2025-098980)
Supplement: online supplemental file 1 [file bmjopen-15-11-s001.docx]

| **Supplementary Table 1. Definition of the predictor variables. (DHS, 2011-22)** | |
| --- | --- |
| **Level of the variable** | **Definition** |
| **Country-level** | |
| GDP per capita | Based on purchasing power parity (current international dollars), obtained from the World Bank database and expressed on a logarithmic scale |
| **Province-level** | |
| ***Urbanization, age composition, and income*** | |
| Level of urbanization | Proportion of household members who are living in an urban area |
| Women’s age | Mean women’s age |
| Absolute income | Median annual household absolute income (in 2017 purchasing power parity–adjusted US dollars) |
| ***Women’s empowerment*** | |
| Education | Mean years of schooling completed by women aged 15–49 |
| Employment | Proportion of women aged 15–49 currently employed |
| SWPER (2 domains) | Percentage of women aged 15-49 with high empowerment in the attitude to violence domain  Percentage of women aged 15-49 with high empowerment in the decision-making domain |
| ***Gender disparity*** | |
| Male-to-female educational attainment ratio | Ratio of men to women aged 15–49 with completed secondary or higher education |
| ***Social practices*** | |
| Early marriage | Percentage of women aged 20-24 years who were first married or in union before age 18 |
| Adolescent childbearing | Percentage of women aged 15-19 who have begun childbearing |

| **Supplementary Table 2. List of countries included in the analyses (LMIC). mDFPS, SII, and CIX are median estimates across provinces from each country. N = 46 countries** | | | | | | |  |
| --- | --- | --- | --- | --- | --- | --- | --- |
| **Countries** | **Survey year** | **Income group** | **N of provinces** | **Median mDFPS (%)** | **Median SII** | **Median CIX** | |
| Albania | 2017 | Middle | 12 | 6.7 | -0.3 | 1.0 | |
| Angola | 2015 | Middle | 18 | 10.6 | 34.0 | 42.4 | |
| Armenia | 2015 | Middle | 11 | 31.6 | 13.6 | 6.5 | |
| Benin | 2017 | Low | 12 | 26.6 | 10.8 | 8.9 | |
| Burkina Faso | 2021 | Low | 13 | 64.4 | 12.1 | 2.9 | |
| Burundi | 2016 | Low | 18 | 35.0 | 13.0 | 8.0 | |
| Cambodia | 2021 | Middle | 25 | 61.5 | -5.4 | -1.9 | |
| Cameroon | 2018 | Middle | 12 | 34.6 | 26.4 | 11.6 | |
| Chad* | 2014 | Low | 19 | 7.4 | 11.0 | 22.1 | |
| Comoros | 2012 | Low | 3 | 24.7 | 17.8 | 15.4 | |
| DRC | 2013 | Low | 11 | 13.7 | 13.6 | 24.6 | |
| Cote d’Ivoire | 2021 | Middle | 14 | 39.1 | 25.8 | 11.0 | |
| Dominican Republic | 2013 | Middle | 9 | 83.6 | 6.7 | 1.3 | |
| Ethiopia | 2016 | Low | 11 | 57.6 | 51.2 | 17.8 | |
| Gabon | 2019 | Middle | 11 | 24.2 | 12.0 | 11.0 | |
| Gambia | 2019 | Low | 8 | 40.1 | 4.2 | 2.4 | |
| Ghana | 2022 | Middle | 16 | 44.7 | -5.2 | -1.5 | |
| Guatemala | 2014 | Middle | 8 | 66.9 | 27.0 | 6.8 | |
| Guinea | 2018 | Low | 8 | 13.3 | 19.0 | 33.8 | |
| Haiti | 2016 | Low | 11 | 43.4 | 5.2 | 2.5 | |
| Honduras | 2011 | Middle | 18 | 74.5 | 11.2 | 2.7 | |
| India | 2019 | Middle | 36 | 71.4 | -1.6 | -0.3 | |
| Indonesia | 2017 | Middle | 34 | 73.8 | -10.0 | -2.8 | |
| Kenya | 2022 | Middle | 47 | 72.4 | 3.7 | 0.8 | |
| Kyrgyzstan | 2012 | Low | 9 | 63.7 | -8.7 | -2.6 | |
| Lesotho | 2014 | Middle | 10 | 74.9 | 18.4 | 4.1 | |
| Liberia | 2019 | Low | 5 | 44.0 | 6.6 | 2.5 | |
| Madagascar | 2021 | Low | 23 | 62.6 | 1.2 | -0.6 | |
| Malawi | 2015 | Low | 3 | 71.6 | 8.3 | 2.0 | |
| Mali | 2018 | Low | 9 | 32.6 | 27.3 | 13.8 | |
| Mauritania | 2019 | Middle | 14 | 26.3 | 13.0 | 8.8 | |
| Myanmar | 2015 | Middle | 15 | 71.7 | 8.3 | 1.8 | |
| Namibia | 2013 | Middle | 13 | 73.4 | 21.9 | 5.5 | |
| Nepal | 2022 | Middle | 7 | 55.1 | -7.9 | -2.5 | |
| Nigeria | 2018 | Middle | 6 | 29.0 | 22.2 | 11.9 | |
| Papua New Guinea | 2016 | Middle | 4 | 47.7 | 19.9 | 6.9 | |
| Rwanda | 2019 | Low | 5 | 71.7 | -10.1 | -2.2 | |
| Senegal | 2019 | Middle | 14 | 47.4 | 13.9 | 5.0 | |
| Sierra Leone | 2019 | Low | 5 | 46.6 | 18.0 | 6.8 | |
| South Africa | 2016 | Middle | 9 | 76.9 | 3.8 | 0.6 | |
| Tanzania | 2022 | Middle | 31 | 49.2 | 11.4 | 3.2 | |
| Timor Leste | 2016 | Middle | 13 | 48.5 | 9.2 | 3.4 | |
| Togo | 2013 | Low | 6 | 32.6 | 3.9 | 2.1 | |
| Uganda | 2016 | Low | 15 | 52.0 | 17.5 | 6.9 | |
| Zambia | 2018 | Middle | 10 | 67.8 | 19.4 | 5.0 | |
| Zimbabwe | 2015 | Low | 10 | 84.9 | 15.0 | 2.6 | |
| DRC: Democratic Republic of the Congo.  ******The provinces of Barh El Gazal and Hadjer Lamis were excluded from the analysis due to the inability to calculate the male-to-female educational attainment ratio, as no women in these provinces had completed secondary or higher education.* | | | | | | |  |

| **Supplementary Table 3. Pearson’s correlation coefficients (in bold) and p-values at the province level. N=621 provinces** | | | | | | | | | | | | | | |  |
| --- | --- | --- | --- | --- | --- | --- | --- | --- | --- | --- | --- | --- | --- | --- | --- |
| **Variables** | **(1)** | **(2)** | **(3)** | **(4)** | **(5)** | **(6)** | **(7)** | **(8)** | **(9)** | **(10)** | **(11)** | **(12)** | **(13)** | **(14)** | |
| (1) mDFPS coverage (%) | **1.000** |  |  |  |  |  |  |  |  |  |  |  |  |  | |
|  |  |  |  |  |  |  |  |  |  |  |  |  |  |  | |
| (2) SII mDFPS | **-0.318** | **1.000** |  |  |  |  |  |  |  |  |  |  |  |  | |
|  | (<0.001) |  |  |  |  |  |  |  |  |  |  |  |  |  | |
| (3) CIX mDFPS | **-0.572** | **0.762** | **1.000** |  |  |  |  |  |  |  |  |  |  |  | |
|  | (<0.001) | (<0.001) |  |  |  |  |  |  |  |  |  |  |  |  | |
| (4) Urban population (%) | **-0.010** | **-0.121** | **-0.079** | **1.000** |  |  |  |  |  |  |  |  |  |  | |
|  | (0.798) | (0.004) | (0.062) |  |  |  |  |  |  |  |  |  |  |  | |
| (5) Mean women's age (years) | **0.303** | **-0.434** | **-0.353** | **0.080** | **1.000** |  |  |  |  |  |  |  |  |  | |
|  | (<0.001) | (<0.001) | (<0.001) | (0.047) |  |  |  |  |  |  |  |  |  |  | |
| (6) Median absolute income (US$) | **0.132** | **-0.178** | **-0.180** | **0.666** | **0.387** | **1.000** |  |  |  |  |  |  |  |  | |
|  | (0.001) | (<0.001) | (<0.001) | (<0.001) | (<0.001) |  |  |  |  |  |  |  |  |  | |
| (7) Mean women's schooling (years) | **0.392** | **-0.384** | **-0.418** | **0.393** | **0.513** | **0.548** | **1.000** |  |  |  |  |  |  |  | |
|  | (<0.001) | (<0.001) | (<0.001) | (<0.001) | (<0.001) | (<0.001) |  |  |  |  |  |  |  |  | |
| (8) Women currently employed (%) | **-0.017** | **-0.065** | **0.037** | **-0.098** | **-0.057** | **-0.283** | **-0.208** | **1.000** |  |  |  |  |  |  | |
|  | (0.677) | (0.121) | (0.383) | (0.014) | (0.154) | (<0.001) | (<0.001) |  |  |  |  |  |  |  | |
| (9) High SWPER attitude to violence (%) | **0.215** | **-0.171** | **-0.210** | **0.440** | **0.219** | **0.500** | **0.452** | **-0.141** | **1.000** |  |  |  |  |  | |
|  | (<0.001) | (<0.001) | (<0.001) | (<0.001) | (<0.001) | (<0.001) | (<0.001) | (<0.001) |  |  |  |  |  |  | |
| (10) High SWPER decision-making (%) | **0.385** | **-0.329** | **-0.335** | **0.153** | **0.418** | **0.274** | **0.594** | **-0.148** | **0.368** | **1.000** |  |  |  |  | |
|  | (<0.001) | (<0.001) | (<0.001) | (<0.001) | (<0.001) | (<0.001) | (<0.001) | (<0.001) | (<0.001) |  |  |  |  |  | |
| (11) High SWPER social independence (%) | **0.368** | **-0.418** | **-0.428** | **0.405** | **0.614** | **0.600** | **0.858** | **-0.221** | **0.411** | **0.609** | **1.000** |  |  |  | |
|  | (<0.001) | (<0.001) | (<0.001) | (<0.001) | (<0.001) | (<0.001) | (<0.001) | (<0.001) | (<0.001) | (<0.001) |  |  |  |  | |
| (12) M/F educational attainment ratio | **-0.465** | **0.183** | **0.293** | **-0.168** | **-0.296** | **-0.255** | **-0.564** | **0.146** | **-0.332** | **-0.512** | **-0.483** | **1.000** |  |  | |
|  | (<0.001) | (<0.001) | (<0.001) | (<0.001) | (<0.001) | (<0.001) | (<0.001) | (<0.001) | (<0.001) | (<0.001) | (<0.001) |  |  |  | |
| (13) Early marriage (%) | **-0.337** | **0.369** | **0.442** | **-0.316** | **-0.440** | **-0.405** | **-0.705** | **0.082** | **-0.360** | **-0.533** | **-0.803** | **0.475** | **1.000** |  | |
|  | (<0.001) | (<0.001) | (<0.001) | (<0.001) | (<0.001) | (<0.001) | (<0.001) | (0.040) | (<0.001) | (<0.001) | (<0.001) | (<0.001) |  |  | |
| (14) Adolescent childbearing (%) | **-0.266** | **0.394** | **0.474** | **-0.187** | **-0.524** | **-0.413** | **-0.534** | **0.185** | **-0.202** | **-0.384** | **-0.656** | **0.329** | **0.709** | **1.000** | |
|  | (<0.001) | (<0.001) | (<0.001) | (<0.001) | (<0.001) | (<0.001) | (<0.001) | (<0.001) | (<0.001) | (<0.001) | (<0.001) | (<0.001) | (<0.001) |  | |
