# Supplementary material for: Influence of subnational contextual factors on demand for family planning satisfied by modern methods: a multilevel approach in 46 surveys from low- and middle-income countries
Source: BMJ Open. 2025 Nov 23;15(11):e098980. doi: 10.1136/bmjopen-2025-098980 (PMC12645598; doi:10.1136/bmjopen-2025-098980)
Supplement: Supplementary file 2 [file bmjopen-2025-098980.reviewer_comments.pdf]

PEER REVIEW HISTORY

BMJ Open publishes all reviews undertaken for accepted manuscripts. Reviewers are asked to complete a checklist review form and are provided with free text boxes to elaborate on their assessment. These free text comments are reproduced below.

ARTICLE DETAILS

Title (Provisional)

Influence of subnational contextual factors on demand for family planning satisfied by modern methods: a multilevel approach in 46 surveys from low- and middle-income countries

Authors

Lima, Natália P; Blumenberg, Cauane; Hellwig, Franciele; Barros, Aluisio JD; Wehrmeister, Fernando C

VERSION 1 - REVIEW

**Reviewer**	**1**
**Name**	**Baia, Ines**
**Affiliation**	**ProChild CoLab Against Poverty and Social Exclusion - Association**
**Date**	**09-May-2025**
**COI**	**None**

I would like to congratulate the authors for this important work. In my opinion the paper is a first and important step to really think about interventions and policies that address family planning, women's empowerment and gender equality in education in low- and middle-income countries.

I would only suggest that authors could explore a little more in the introduction the modern methods, some information on their current use and accessibility in these countries, in order to then contextualize the results and discussion.

**Reviewer**	**2**
**Name**	**Pinho-Gomes, Ana-Catarina**
**Affiliation**	**University of Oxford, The George Institute for Global Health**
**Date**	**14-Jul-2025**

COI	None

Lima et all undertook an observational study investigating the association between province-level contextual factors and demand for family planning satisfied by modern methods (mDFPS) in LMICs. They demonstrated that women's education and employment were positively associated with greater demand for mDFPS whilst larger gender imbalances in education were negatively associated with demand for mDFPS. Their study illustrates how women's empowerment and gender equality may increase use of mDFPS, which is beneficial for women's health and wellbeing. There are important limitations, specifically the ecological nature of this study and the inclusion of only married women. These limitations, acknowledged by the authors, limit not only the generalisability but also the strength and applicability of the evidence. Although the novelty of the study findings is questionable, it is an interesting paper that strengthens the evidence on the importance of gender equality by adding another important dimension of reproductive health.

Page 5, line 5

Contraception is not just a basic right. It is an essential way to ensure reproductive health and women's wellbeing as multiple pregnancies and abortions, especially if not adequately spaced in time, pose significant risk to women's health.

Page 10

I would suggest organising the results by LIC and MIC instead of by predictive factor as currently it is very confusing and difficult to grasp the key messages/findings of this study. It would also help to identify the differences between LICs and MICs.

Page 14, line 28

The ecological study design not only limits the ability to make causal inferences but is also susceptible to ecological bias, i.e., the inability to extend the associations found at country level to individual level.

It would be worth adding as limitation the fact that this study did not take into account many important variables that are known to influence, e.g., cultural beliefs, healthcare system and public health system development.

I would also suggest adding as limitation the significant correlation between the predictive factors tested by this study. We have previously found that gender inequality is associated with better women's life expectancy, which is heavily influenced by maternal mortality, which is itself dependent on access to and use of contraceptives.

Page 14, line 45

I doubt that a country level analysis is not superior to community level analysis. The smaller and closer to the individual level of analysis, the more likely that findings are accurate.

County level analyses have a substantial risk of ecological bias. I suggest the authors remove that as a strength.

It would be of interest to test validated indices of inequality, such as the UN gender inequality index to further support the findings of this study.

To further support the importance of this analysis, it would be interesting to test the association with contraceptive use is maternal and child mortality and abortion rate as these are likely affected by unwanted and inadequately spaced pregnancies. Could this be added maybe?

**Reviewer**	**3**
**Name**	**Barry, Sarah J.E.**
**Affiliation**	**Frontier Science Scotland Ltd, Biostatistics**
**Date**	**02-Sep-2025**
**COI**	**None**

This is a rigorously conducted study and I only have minor comments.

SWPER - could the authors add a footnote to the tables where this is included to remind the reader that high SWPER represents high empowerment.

The authors should add either a footnote or a sentence to the legends of Tables 2 and 3 to explain how to interpret the betas.

VERSION 1 - AUTHOR RESPONSE

Reviewer: 1
Ms. Ines Baia, ProChild CoLab Against Poverty and Social Exclusion - Association
Comments to the Author:
I would like to congratulate the authors for this important work. In my opinion the paper is a first and important step to really think about interventions and policies that address family planning, women's empowerment and gender equality in education in low- and middle-income countries.
I would only suggest that authors could explore a little more in the introduction the modern methods, some information on their current use and accessibility in these countries, in order to then contextualize the results and discussion.

We thank the reviewer for the very positive feedback. We agree with the suggestion and have now expanded the introduction to include additional information on modern contraceptive methods, accessibility, and use in LMICs.

Reviewer: 2
Dr. Ana-Catarina Pinho-Gomes, University of Oxford

Comments to the Author:
Lima et all undertook an observational study investigating the association between province-level contextual factors and demand for family planning satisfied by modern methods (mDFPS) in LMICs. They demonstrated that women's education and employment were positively associated with greater demand for mDFPS whilst larger gender imbalances in education were negatively associated with demand for mDFPS. Their study illustrates how women's empowerment and gender equality may increase use of mDFPS, which is beneficial for women's health and wellbeing. There are important limitations, specifically the ecological nature of this study and the inclusion of only married women. These limitations, acknowledged by the authors, limit not only the generalisability but also the strength and applicability of the evidence. Although the novelty of the study findings is questionable, it is an interesting paper that strengthens the evidence on the importance of gender equality by adding another important dimension of reproductive health.

We thank the reviewer for the careful reading and insightful comments, which have helped us improve the clarity and robustness of our manuscript. Below we address each point in detail.

Page 5, line 5
Contraception is not just a basic right. It is an essential way to ensure reproductive health and women's wellbeing as multiple pregnancies and abortions, especially if not adequately spaced in time, pose significant risk to women's health.

Thank you for your comment. We agree with your observation that contraception plays a crucial role not only as a basic right but also in protecting women's health by reducing the risks associated with multiple and closely spaced pregnancies. We believe this point is already addressed in the current version of the manuscript, where we discuss how contraceptive use prevents unintended pregnancies, reduces closely spaced births, and consequently decreases high-risk pregnancies, poor perinatal outcomes, and maternal and child mortality. Please let us know if further clarification is needed.

Page 10
I would suggest organising the results by LIC and MIC instead of by predictive factor as currently it is very confusing and difficult to grasp the key messages/findings of this study. It would also help to identify the differences between LICs and MICs.

We reorganized the results by country income level (LICs vs MICs), as suggested.

Page 14, line 28
The ecological study design not only limits the ability to make causal inferences but is also susceptible to ecological bias, i.e., the inability to extend the associations found at country level to individual level.

Thank you for your valuable comment. We have expanded the discussion to acknowledge ecological bias as a limitation of the ecological analysis. Additionally, we highlighted that our multilevel analysis, which includes individual-level data, provided results consistent with the ecological findings, helping to mitigate concerns related to ecological bias.

It would be worth adding as limitation the fact that this study did not take into account many important variables that are known to influence, e.g., cultural beliefs, healthcare system and public health system development.

We expanded our discussion of limitations to emphasize the absence of important contextual variables, such as cultural norms, the healthcare system and public health system development, especially at the provincial level.

I would also suggest adding as limitation the significant correlation between the predictive factors tested by this study. We have previously found that gender inequality is associated with better women's life expectancy, which is heavily influenced by maternal mortality, which is itself dependent on access to and use of contraceptives.

Thank you for your insightful comment. We recognize the complex relationships between gender inequality, maternal mortality, and family planning in a broader context. To address potential multicollinearity among predictor variables, we tested their correlations using Pearson's correlation coefficients and excluded variables with high collinearity ($r > 0.8$) to ensure model robustness within the scope of this study. This procedure has been detailed in the methods section.

Page 14, line 45
I doubt that a country level analysis is not superior to community level analysis. The smaller and closer to the individual level of analysis, the more likely that findings are accurate. County level analyses have a substantial risk of ecological bias. I suggest the authors remove that as a strength.

Thank you for your comment. We agree that analyses at smaller and more proximate levels can offer more accurate estimates. In our revision, we clarified in the manuscript that while such approaches may be more accurate, focusing on provinces allows us to capture broader contextual factors that are relevant for informing region-specific strategies. Our intention was not to suggest provincial-level analysis is superior, but to highlight that it remains underexplored in multi-country research and offers complementary insights into community- and country-level analyses.

It would be of interest to test validated indices of inequality, such as the UN gender inequality index to further support the findings of this study.

Thank you for the thoughtful suggestion. While indices such as the UN Gender Inequality Index offer valuable perspectives, our study focused on examining specific contextual factors at the subnational level. Unfortunately, most of these indices are calculated only at the national level and are therefore not applicable to our province-level analysis. We have now included this clarification in the discussion section.

To further support the importance of this analysis, it would be interesting to test the association with contraceptive use is maternal and child mortality and abortion rate as these are likely affected by unwanted and inadequately spaced pregnancies. Could this be added maybe?

We thank the reviewer for highlighting the relevance of maternal and child mortality and abortion rates, which are indeed closely related to contraceptive use. However, addressing these outcomes would require a different analytical approach and research design. For this reason, we deliberately focused on demand for family planning satisfied, a well-established and widely comparable indicator of contraceptive coverage. We acknowledge, however, that future research could expand on this important topic by linking contraceptive use with maternal and child health outcomes.

Reviewer: 3
Dr. Sarah J.E. Barry, Frontier Science Scotland Ltd
Comments to the Author:
This is a rigorously conducted study and I only have minor comments.

SWPER - could the authors add a footnote to the tables where this is included to remind the reader that high SWPER represents high empowerment.

The authors should add either a footnote or a sentence to the legends of Tables 2 and 3 to explain how to interpret the betas.

We appreciate the reviewer's positive feedback and helpful suggestions. We have added a footnote to the tables where the SWPER index is included, indicating that higher SWPER scores reflect higher women's empowerment. In addition, we added a footnote in Tables 2 and 3 clarifying that beta coefficients represent the change in the outcome associated with a one-unit increase in the predictor; positive values indicate a direct association, while negative values indicate an inverse association.

VERSION 2 - REVIEW

**Reviewer**	**2**
**Name**	**Pinho-Gomes, Ana-Catarina**
**Affiliation**	**University of Oxford, The George Institute for Global Health**
**Date**	**29-Oct-2025**
**COI**	

Thanks for addressing my comments and congratulations on your paper.
